# Serum vitamin D concentrations in hospitalized critically ill dogs

**DOI:** 10.1371/journal.pone.0194062

**Published:** 2018-03-28

**Authors:** Jared A. Jaffey, Robert C. Backus, Kaylyn M. McDaniel, Amy E. DeClue

**Affiliations:** Department of Veterinary Medicine and Surgery, Veterinary Health Center, University of Missouri, Columbia, Missouri, United States of America; National Yang-Ming University, TAIWAN

## Abstract

Hypovitaminosis D has been extensively documented in critically ill humans. However, whether or not critically ill dogs have alterations in vitamin D concentrations remains unconfirmed. The primary aims of our study were to compare serum 25-hydroxycholecalciferol [25(OH)D] concentrations in critically ill dogs with healthy control dogs, determine the prognostic utility of serum 25(OH)D concentration as a biomarker in critically ill dogs, and to assess if serum 25(OH)D concentrations in critically ill dogs are associated with length of stay in the intensive care unit or illness severity. Serum concentrations of 25(OH)D together with a range of other clinical, biochemical, and hematological parameters, were measured in 99 dogs within 24 hours of admission to the Intensive Care Unit (ICU). Critically ill dogs (P = 0.001) and dogs with sepsis (P = 0.002) had significantly lower serum 25(OH)D concentrations compared to healthy control dogs. In addition, serum 25(OH)D concentration was an independent predictor of in-hospital and 30 day survival. Using a cut-off of 33 ng/mL, serum 25(OH)D concentrations had excellent sensitivity (0.94; 95% CI, 0.71–1.00), but poor specificity (0.41; 95% CI, 0.31–0.53) for detection of survival. Serum 25(OH)D concentrations were inversely associated with acute patient physiologic and laboratory evaluation (APPLE) fast score but were not associated with ICU length of stay. Hospitalized dogs with critical illness have decreased serum 25(OH)D concentrations compared to healthy dogs and can be used to predict survival in this cohort.

## Introduction

Hypovitaminosis D has been extensively investigated in critically ill people. The prevalence of hypovitaminosis D in critically ill people is as high as 82% [1] and is associated with increased mortality [1–9], length of hospitalization [1–10], sepsis [8,9,11,12], and illness severity [13]. While vitamin D is classically thought of in the context of calcium, phosphorous, and skeletal homeostasis [14–16], identification of vitamin D receptors and 1-alpha hydroxylase in tissues outside of the kidney suggests more extensive effects than previously thought [16]. The pleiotropic effects of vitamin D extend to regulation of cell proliferation, angiogenesis, blood pressure, as well as the innate and adaptive immune systems [17].

The importance of hypovitaminosis D in the context of critical illness may relate to its immunomodulatory properties. Dogs with naturally developing critical illness might be an ideal pre-clinical model for studying hypovitaminosis D during critical illness in people since dogs have similar environmental exposures, co-morbidities and undergo similar medical and surgical interventions. Further, vitamin D augments the innate immune response in people by increasing phagocytosis [18–20] and induction of antimicrobial peptides [21–25] as well as decreases leukocyte production of pro-inflammatory cytokines such as tumor necrosis factor (TNF)-α and interleukin (IL)-6 while increasing production of the anti-inflammatory cytokine IL-10 [26–29]. Likewise, vitamin D blunts leukocyte production of TNF without compromising toll-like receptor 4 expression or phagocytosis in neutrophils and monocytes in dogs [30]. In addition, vitamin D increases lipopolysaccharide stimulated leukocyte production of IL-10 in endotoxin primed whole blood in dogs, in vitro (Jaffey et al., unpublished work) further suggesting the dog has similarities to people. Dogs are not able to synthesize adequate amounts of vitamin D in response to ultraviolet light from the sun, and thus are dependent on dietary intake to meet their vitamin D needs [31]. Therefore, studies assessing the effects of vitamin D supplementation in dogs will not be limited by confounding variables (e.g., latitude [32], seasonality [33], age [33], ethnicity [34], obesity [35], or gender [36]) inherent to similar studies in humans.

The primary aims of our study were to determine if dogs, like people, have decreased serum concentrations of vitamin D during critical illness and to determine if decreased serum concentrations of vitamin D were associated with illness severity and outcome. We hypothesized that critically ill dogs and dogs with sepsis would have significantly lower serum 25-hydroxycholecalciferol [25(OH)D] concentrations than healthy control dogs. Further, we hypothesized that critically ill dogs with higher serum 25(OH)D concentrations would be more likely to survive to discharge and be alive 30 days after discharge. Secondary aims were to compare serum 25(OH)D concentrations in dogs with sepsis and healthy control dogs as well as to compare serum 25(OH)D concentrations in dogs with sepsis that survived to discharge with those that did not survive. We hypothesized that dogs with sepsis would have lower serum 25(OH)D concentrations than healthy control dogs and that dogs with sepsis that survived to discharge would have greater serum 25(OH)D concentrations than dogs that did not survive.

## Materials and methods

Dogs admitted to the University of Missouri Veterinary Health Center Intensive Care Unit (ICU) between October 2016 and January 2017 were eligible for inclusion in this prospective observational study. Client consent was obtained. This study was conducted in accordance with guidelines for clinical studies and was approved by the University of Missouri Animal Care and Use Committee (protocol 7334). Exclusion criteria dogs included, pregnancy, lactating, hypercalcemia of malignancy, hyperparathyroidism, hypoparathyroidism, known history of chronic kidney disease, supplementation with vitamin D or calcium, and if a dog had already been admitted to the ICU during the study period. Furthermore, dogs were excluded if blood glucose, albumin, lactate, platelet count, and mentation score were not obtained within 24 hours of ICU admission in order to determine the acute patient physiologic and laboratory evaluation (APPLE) fast score for each dog. A second population of dogs were also enrolled as a control population. The control dogs were determined to be healthy based on history and physical examination. Control dogs could not have any illnesses, vaccination or administration of medications, with the exception of routine parasitic prevention, within 30 days of enrollment.

The APPLE fast scoring system was used to assess illness severity. The APPLE fast score for each dog was calculated using blood glucose, albumin, lactate, platelet count, and mentation score obtained within 24 hours of admission to the ICU (Table 1) [37]. Criteria for mentation scoring are summarized in (Table 2). All mentation scores were performed by a single individual (JAJ). Dogs were considered to have sepsis if they met 2 or more of the criteria for systemic inflammatory response syndrome (heart rate ≥160 bpm, respiratory rate ≥ 40 bpm, temperature ≤ 37.8 or ≥ 39.7°C, and white blood cell count ≤ 4 or ≥ 12 x 10^3^/μL or ≥ 10% band neutrophils) [38] and had either cytological or microbiological confirmation of bacterial infection or related to intestinal bacterial translocation.

**Table 1:** APPLE fast score. Calculated by adding the values in the upper left corner of each cell for the 5 parameters listed, with a maximum score of 50 [37]. Adapted from Hayes et al., 2010.

***7***	***8***	***9***	***10***	**Glucose (mg/dL)**				
**< 84**	**84–102**	**103–164**	**165–273**	**> 273**				
	***8***	***7***	***6***	**Albumin (g/dL)**	**2**			
	**< 2.6**	**2.6–3.0**	**3.1–3.2**	**3.3–3.5**	**> 3.5**			
				**Lactate (mmol/L)**	***4***	***8***	***12***	
				**< 1.0**	**1.0–4.0**	**4.1–5.0**	**> 5.0**	
	***5***	***6***	***3***	**Platelet count (X 10**^**3**^**/**μ**l)**	***1***			
	**< 151**	**151–200**	**201–260**	**261–420**	**> 420**			
				**Mentation score**	***4***	***6***	***7***	***14***
				**0**	**1**	**2**	**3**	**4**

**Table 2:** Mentation score [37]. Adapted from Hayes et al., 2010.

Score	Definition
0	Normal
1	Able to stand unassisted,
	responsive but dull
2	Can stand only when assisted,
	responsive but dull
3	Unable to stand, responsive
4	Unable to stand, unresponsive

### Sample collection

Medical records were reviewed for each dog enrolled. The age, sex and breed were recorded for each dog. The following clinical information was extracted for each dog within 24 hours of ICU admission: white blood cell count, platelet count, albumin, glucose, lactate, and serum 25(OH)D concentrations. In addition, the duration of hospitalization, reason for ICU admission, and survival were recorded. Survival data was obtained from clinical records or follow up telephone calls to clients for each dog for survival to discharge and day 30 after ICU discharge. All hematology and biochemical parameters were measured using the same analyzers. Blood lactate concentrations were measured either on a blood gas analyzer or a handheld device previously validated in dogs [39]. Blood samples were collected into evacuated glass tubes and were centrifuged (1,500 x g, 7 minutes) and serum harvested within 1 hour of sample collection. The serum was placed in an airtight, freezer-resistant plastic tube and stored for a maximum of 4 months at -80°C for batch analysis of serum 25(OH)D concentrations [40]. Serum concentrations of 25(OH)D were determined in thawed serum using a modification of a HPLC method previously reported [41]. Tubes were coded so that the identity of the sample was only known to the investigator and not to the laboratory measuring serum 25(OH)D. In addition, serum 25(OH)D concentrations were evaluated after all dogs had been discharged from the ICU, died, or were euthanized. Therefore, serum 25(OH)D results did not impact the clinical management of the dogs.

### Statistical analyses

Statistical analysis was performed by commercial software. A Shapiro-Wilk test was used to assess normality. The data with a non-normal distribution were assessed using Mann-Whitney *U* test and the median, Q1, Q3, and range given. Categorical variables are presented as proportions. The length of stay was dichotomized based on its median value (3 days) into 2 categories: short stay (≤ 3 days) and long stay (> 3 days). Dogs that died or were euthanized in the ICU were not included in the assessment of length of stay. A receiver-operating characteristic (ROC) curves were used to determine the area under the curve (AUC) and select the optimum cut-off value for detection of death that maximized the Youden’s J statistic for sensitivity and specificity reporting. Multivariate logistic regression analyses were performed for the whole study population, choosing ICU mortality and 30 day post ICU discharge mortality as the dependent variables. Independent variables for the multivariate logistic model were APPLE fast score, serum 25(OH)D concentration, and the presence of sepsis. The correlation between serum 25(OH)D concentration with length of stay and APPLE fast scores were tested by the Spearman test. The level of significance was set at P < 0.05.

## Results

Two-hundred and sixteen dogs fulfilled the inclusions criteria. One-hundred and seventeen dogs were excluded from analysis for the following reasons, lack of hematologic or biochemical data needed to calculate an APPLE fast score (n = 58), owner objection (n = 30), hospitalization for > 24 hours before sample acquisition (n = 11), chronic kidney disease (n = 4), malignancy associated hypercalcemia (n = 4), euthanized before sample acquisition (n = 3), clinician objection (n = 3), primary hyperparathyroidism (n = 2), and 1 each for pregnancy and a dog that had already been enrolled from a previous ICU admission.

### Animal population

Ninety-nine dogs were enrolled. There were 84 purebred dogs and 15 mixed breed dogs included. Breeds were Labrador Retriever (n = 12), Golden Retriever (4), Miniature Dachshund (4), German Shepherd Dog (3), Border Collie (3), Miniature Schnauzer (3), Beagle (2), American Staffordshire Terrier (2), Maltese (2), Jack Russell Terrier (2), Boxer (2), French Bulldog (2), and 1 each of Akita, Australian Shepherd, Bloodhound, Brussels Griffon, Bullmastiff, Chihuahua, Dogue De Bordeaux, Great Dane, Mastiff, Miniature Poodle. The median age at presentation was 7.6 years (Q1, Q3, range; 4.0, 10.6, 0.2–17.5). The median weight was 17.0 kg (7.7, 29.8, 1.6–58.7). There were 48 neutered males, 42 spayed females, 5 intact males, and 4 intact females.

Dogs were admitted to the ICU for the following reasons postoperative supportive care (n = 22); neoplasia (15), septic peritonitis (6), vomiting and diarrhea (5), trauma (5), respiratory distress (4), diabetic ketoacidosis with pancreatitis (3), hypoadrenocorticism (2), gastric dilatation (2), immune-mediated hemolytic anemia (2), seizures of unknown etiology (2), pancreatitis (2), vomiting (2), bacterial pneumonia (3), aspiration pneumonia (3), acute kidney failure (2), and 1 each of subcutaneous abscess, anaphylaxis, cholangiohepatitis, nephrotic syndrome, myxedema coma, diskospondylitis, diabetic ketoacidosis, fever of unknown origin, laryngeal paralysis, unclassified type of pneumonia, lung lobe torsion, lymphangiectasia, meningoencephalitis of unknown origin, pancytopenia and myelodysplasia, protein losing enteropathy of unknown etiology, steroid responsive pneumonia (suspected bronchiolitis obliterans with organizing pneumonia), regurgitation, suspected xylitol toxicity.

A total of 16/99 dogs were diagnosed with sepsis. The source of sepsis was classified as abdominal (8), respiratory (2), unknown (3), and 1 each of following central nervous system, musculoskeletal, and dermatologic/subcutaneous. Infectious agents identified on either cytology, culture, or histopathology included non-specific bacilli (2), rods (2), cocci (1), *Staphylococcus hemolyticus* (2), *Corynebacterium* species (1), *Streptococcus canis* (beta hemolytic Streptococcus)(1), Serratia marcescens (1), *Enterobacter cloacae* (1), and *Enterococcus faecium* (1).

Seventeen of the 99 critically ill dogs died (4) or were euthanized (13) for an in hospital mortality rate of 17.1%. The survival status for dogs 30 days after hospital discharge was available in 93/99 dogs. Twenty-three critically ill dogs died (4) or were euthanized (19) 30 days after discharge for an overall mortality rate of 24.7%. Eight of the 16 dogs with sepsis died or were euthanized in the hospital.

Seventeen healthy control dogs were included. There were 13 purebred dogs and 4 mixed breed dogs. Breeds were Miniature Dachshund (3), Pit-bull Terrier (2), and 1 each of Miniature Poodle, Brittany Spaniel, Catahoula, Labrador Retriever, Golden Retriever, German Shepherd Dog, Pomeranian, Great Dane. The median age was 6.0 years (Q1, Q3, range; 2.5, 9.0, 0.6–12.0). There were 9 spayed females, 6 neutered males, and 2 intact males.

### Serum 25(OH)D concentration and survival

**Fig 1:**
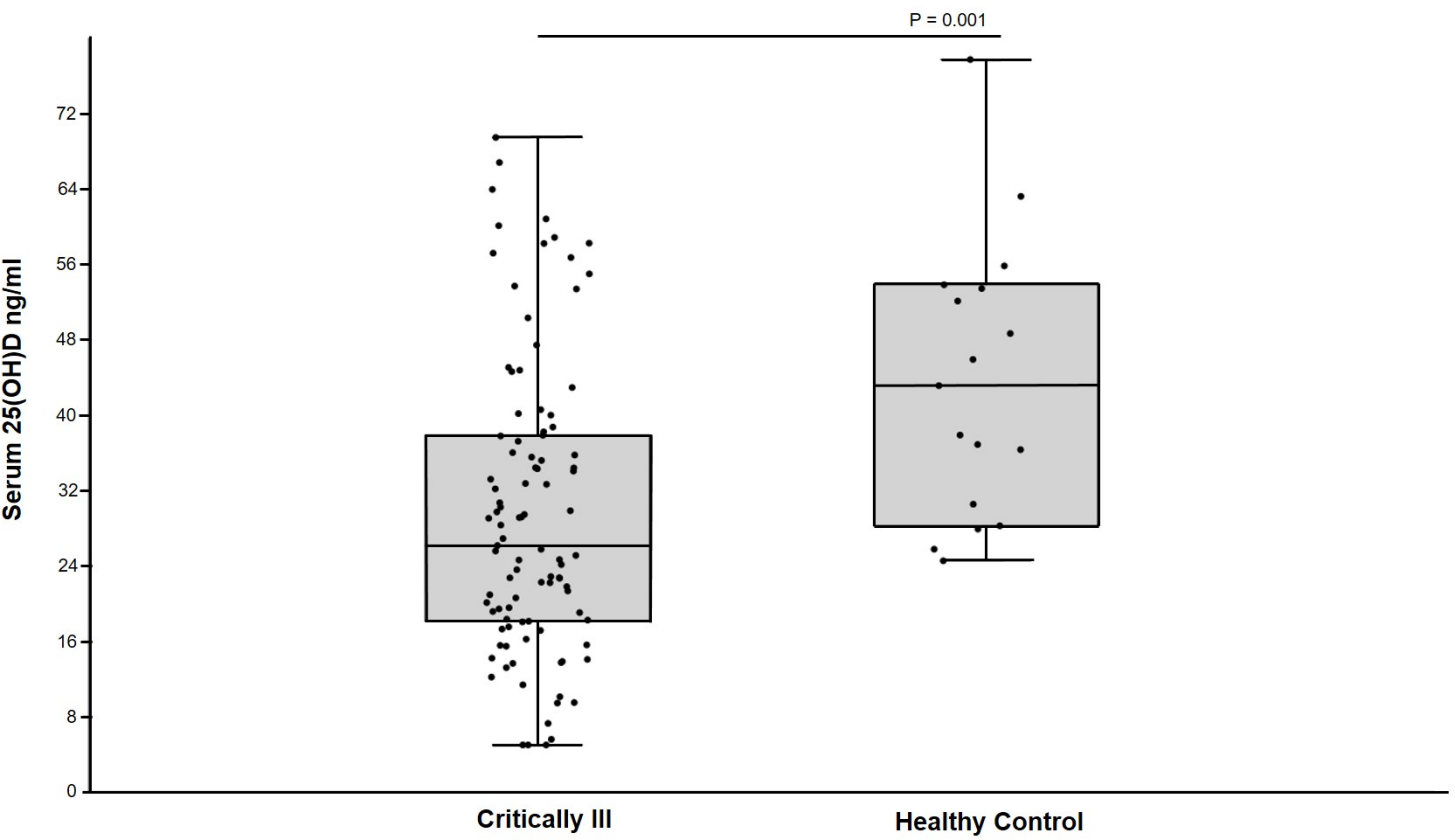
Box and whiskers plot comparing serum 25(OH)D concentrations in critically ill dogs and healthy control dogs. The boxes represent the 25th and 75th quartiles with the horizontal line representing the median. The whiskers represent the range of the data. The black circles represent results for individual dogs. Healthy control dogs (n = 17) had significantly greater serum 25(OH)D concentration than critically ill dogs (n = 99).

**Fig 2:**
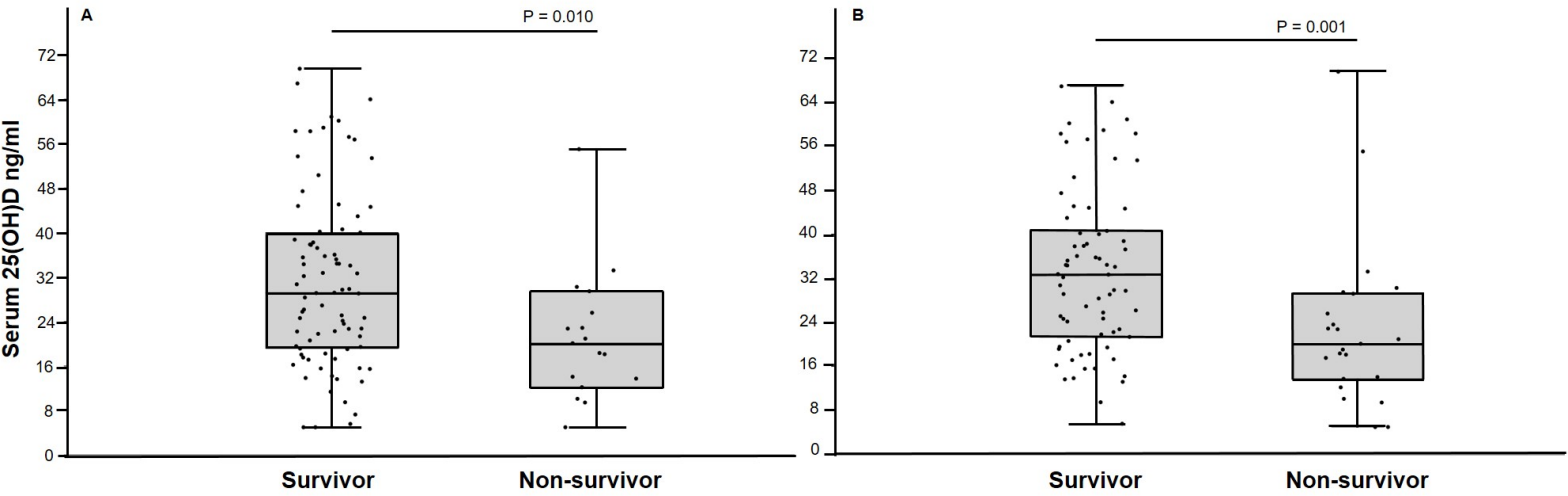
Box and whiskers plot comparing serum 25(OH)D concentrations in survivor and non-survivor critically ill dogs. The boxes represent the 25th and 75th quartiles with the horizontal line representing the median. The whiskers represent the range of the data. The black circles represent results for individual dogs. (A) Dogs that survived (n = 82) to discharge had significantly greater serum 25(OH)D concentration than non-survivors (n = 17). (B) Dogs that were alive (n = 23) 30 days after discharge had significantly greater serum 25(OH)D concentrations than non-survivors (n = 70).

Healthy control dogs had significantly greater serum 25(OH)D concentrations than critically ill dogs (Fig 1). Dogs that survived to discharge had significantly greater serum 25(OH)D concentrations than dogs that died or were euthanized (Fig 2A). Furthermore, dogs that were alive 30 days after discharge had significantly greater serum 25(OH)D concentrations than dogs that were not alive (Fig 2B).

Next, serum 25(OH)D was evaluated in a multivariate logistic regression model to determine if it was an independent predictor of mortality. Diagnosis of sepsis and APPLE fast scores were included in the model as covariates because of their potential association with serum 25(OH)D and mortality. The logistic regression analysis for ICU mortality indicated that only serum 25(OH)D concentrations and sepsis remained significant predictors of survival (Table 3). Importantly, this result indicates that for every 1 ng/ml increase in serum 25(OH)D concentration, the risk for mortality decreased by 6.0%. Serum 25(OH)D concentration was the only independent predictor for survival 30 days after discharge (Table 4). Moreover, for every 1 ng/ml increase in serum 25(OH)D concentration, the risk for mortality 30 days after discharge decreased by 5.0%.

**Table 3:** Multivariate logistic regression model for survival in the intensive care unit.

Variable	Odds ratio (95% CI)	P value
APPLE fast score	0.93 (0.84–1.03)	0.84
Serum 25(OH)D concentration	1.06 (1.00–1.13)	0.046
Sepsis	6.11 (1.35–27.70)	0.02

**Table 4:** Multivariate logistic regression model for survival 30 days after discharge.

Variable	Odds ratio (95% CI)	P value
APPLE fast score	0.92 (0.84–1.01)	0.08
Serum 25(OH)D concentration	1.05 (1.00–1.10)	0.03
Sepsis	4.09 (0.94–17.82)	0.06

**Fig 3:**
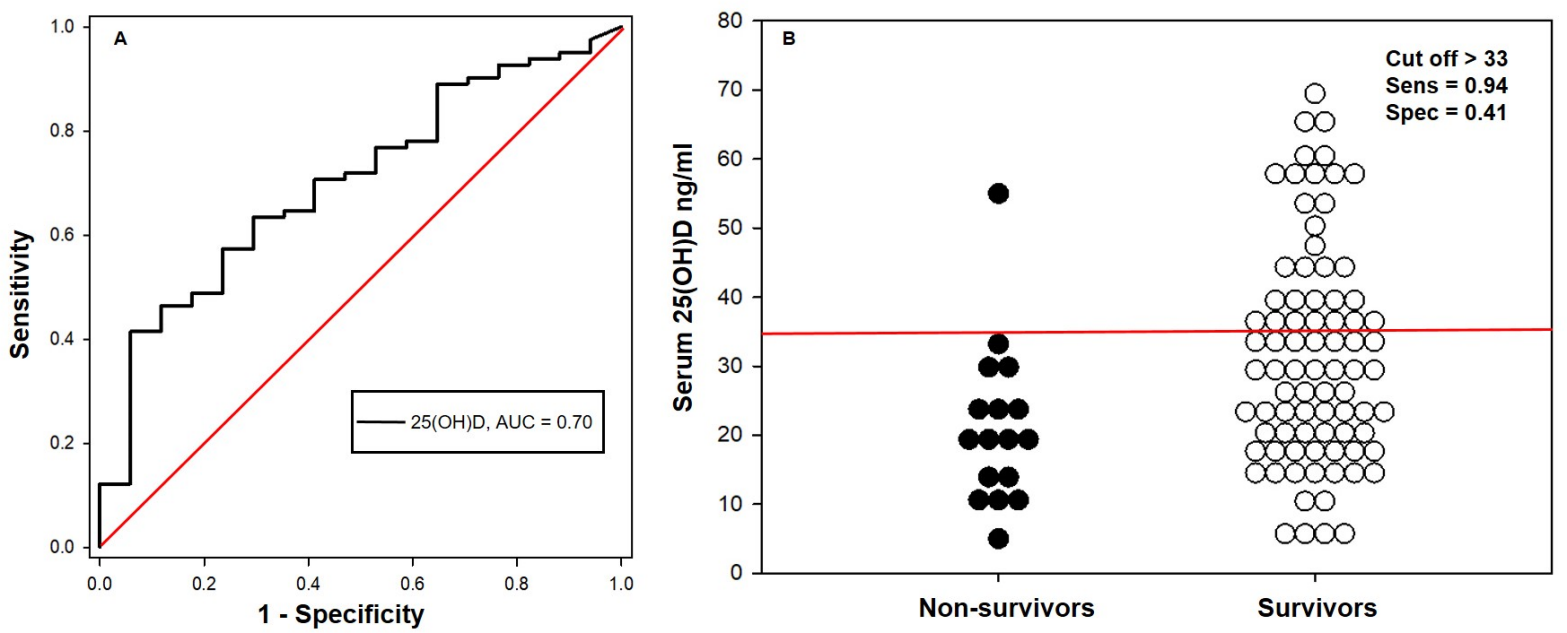
Receiver-operating characteristic (ROC) curve and dot plot comparing the diagnostic sensitivity and 1-specificity of serum 25(OH)D concentration for determining survival in the intensive care unit. (A) The diagonal red line represents the reference line (B) The horizontal red line represents the optimal serum 25(OH)D concentration cutoff of 33 ng/ml.

**Fig 4:**
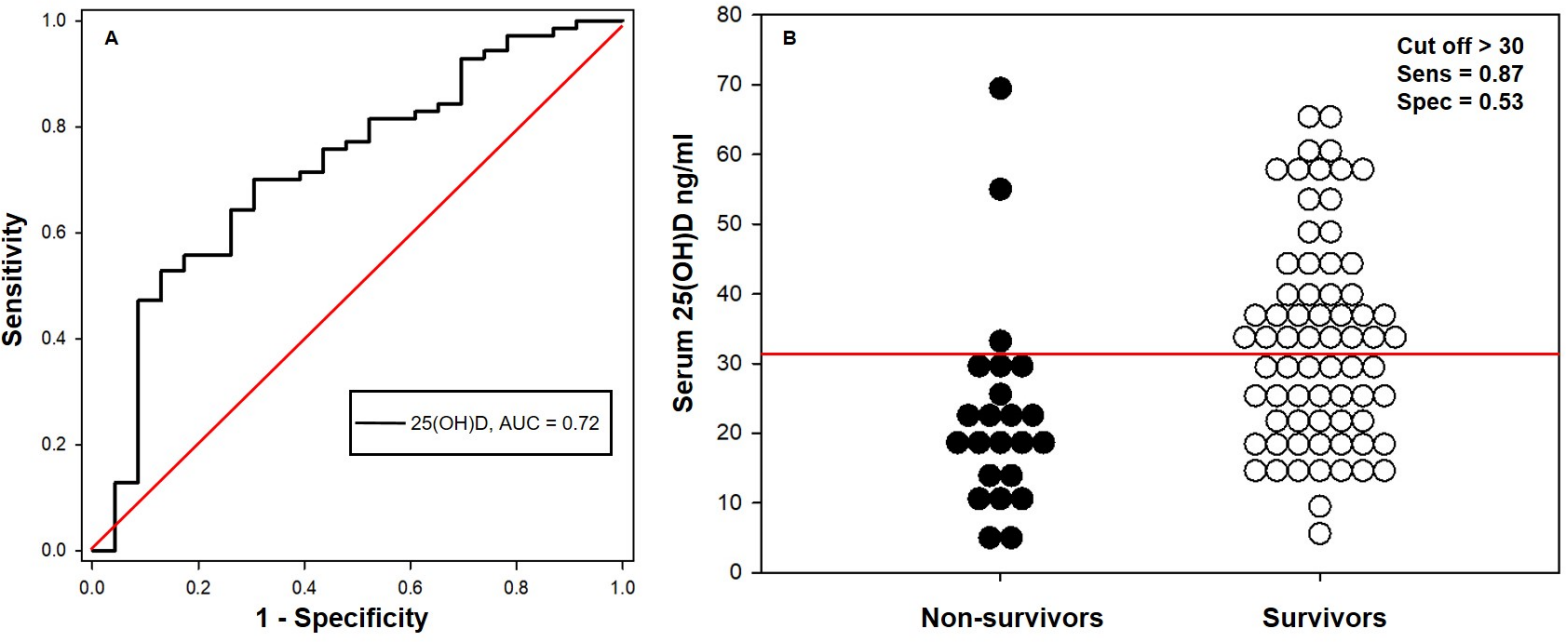
Receiver-operating characteristic (ROC) curve and dot plot comparing the diagnostic sensitivity and 1-specificity of serum 25(OH)D concentration for determining survival 30 days after discharge. (A) The diagonal red line represents the reference line. (B) The horizontal red line represents the optimal serum 25(OH)D concentration cutoff of 30 ng/ml.

The area under the ROC curve for serum 25(OH)D concentrations in relation to ICU survival was 0.70 (95% CI, 0.57–0.83). The optimal cutoff was > 33 ng/ml, with a sensitivity of 0.94 (95% CI, 0.71–1.00) and a specificity of 0.41 (95% CI, 0.31–0.53) for the detection of survival (Fig 3; Table 5). The area under the ROC curve for serum 25(OH)D concentrations in relation to 30 days post ICU discharge survival was 0.72 (95% CI, 0.60–0.84). The optimal cutoff was >30 ng/ml, with a sensitivity of 0.87 (95% CI, 0.66–0.97) and a specificity of 0.53 (95% CI, 0.41–0.65) for detection of survival (Fig 4; Table 6).

**Table 5:** Sensitivity and specificity of serum 25(OH)D concentrations for detection of survival in the ICU using different cutoff values.

Cutoff (ng/ml)	Sensitivity	95% CI	Specificity	95% CI
68	1.00	0.80–1.00	0.02	0.00–0.08
44	0.94	0.71–0.99	0.20	0.12–0.30
38	0.94	0.71–0.99	0.27	0.18–0.38
**33**	**0.94**	**0.71–0.99**	**0.41**	**0.31–0.53**
22	0.65	0.38–0.86	0.63	0.52–0.74
18	0.41	0.18–0.67	0.77	0.66–0.85
13	0.24	0.07–0.50	0.91	0.83–0.97
5	0.06	0.00–0.29	0.98	0.91–1.00

Bold font represents Youdens J statistic

**Table 6:** Cutoff serum 25(OH)D concentrations and resulting sensitivities and specificities for detection of survival 30 days after discharge from the intensive care unit.

Cutoff (ng/ml)	Sensitivity	95% CI	Specificity	95% CI
68	0.96	0.78–1.00	0.00	0–0.05
48	0.91	0.72–0.99	0.17	0.09–0.28
36	0.91	0.72–0.99	0.36	0.25–0.48
**30**	**0.87**	**0.66–0.97**	**0.53**	**0.41–0.65**
25	0.70	0.47–0.87	0.64	0.52–0.75
19	0.48	0.27–0.69	0.81	0.70–0.90
13	0.22	0.07–0.44	0.96	0.88–0.99
5	0.09	0.01–0.28	1.00	0.95–1

Bold font represents Youdens J statistic

### Serum 25(OH)D concentration and ICU length of stay

**Fig 5:**
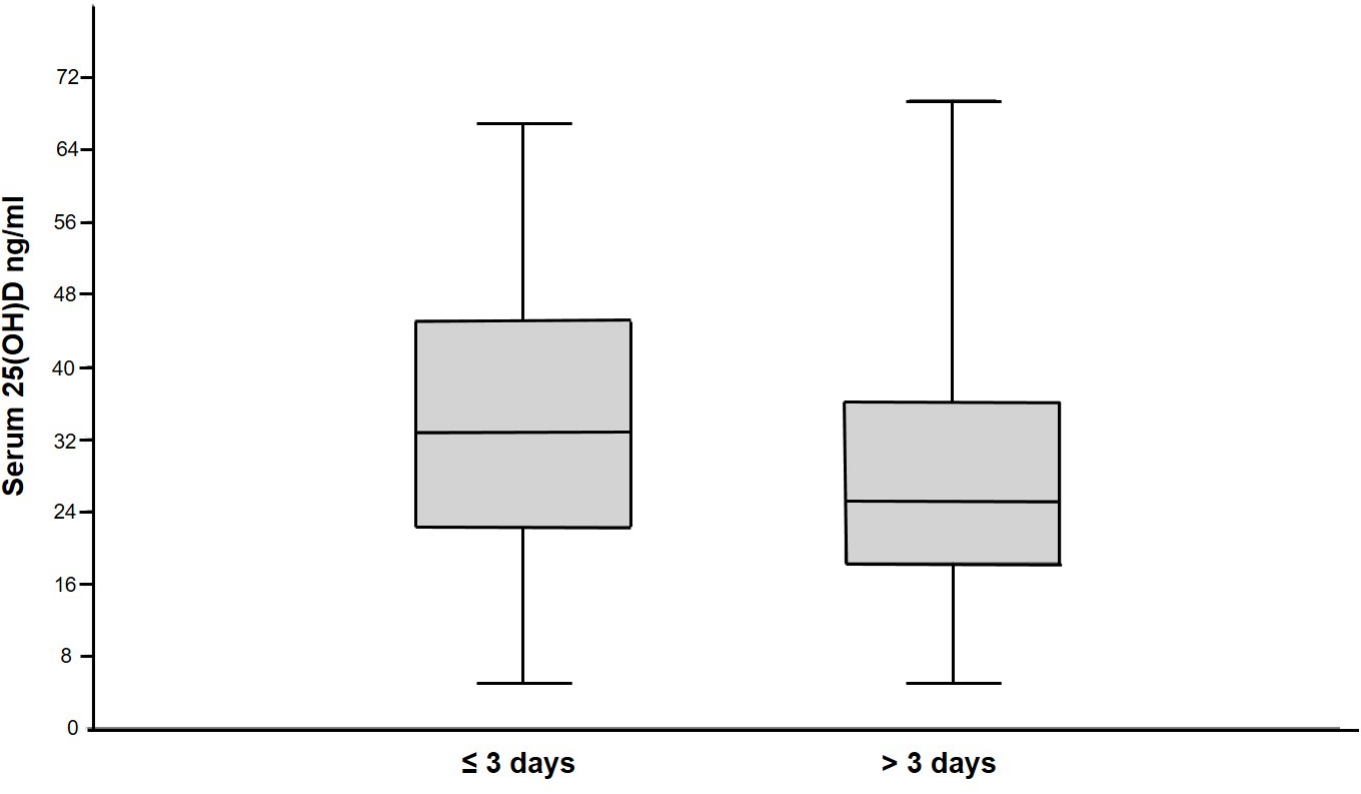
Box and whiskers plot comparing serum 25(OH)D concentrations in dogs that stayed ≤ 3 days in the intensive care unit to those that stayed > 3 days. The boxes represent the 25th and 75th quartiles with the horizontal line representing the median. The whiskers represent the range of the data. There was not a significant difference in serum 25(OH)D concentrations in dogs that stayed ≤ 3 days (n = 42) and those that stayed > 3 days (n = 40, P = 0.14).

The median length of stay for critically ill dogs in the ICU was 3 days (Q1, Q3, range; 3.0, 5.0; 1.0–15). There was not a significant difference in serum 25(OH)D concentrations in dogs that were hospitalized for ≤ 3 days compared to > 3 days (Fig 5). Furthermore, no significant correlation was found between serum 25(OH)D concentration and ICU length of stay when length of stay was analyzed as a continuous variable (n = 82, r_s_ = 0.03, P = 0.79).

### Serum 25(OH)D concentration and illness severity

**Fig 6:**
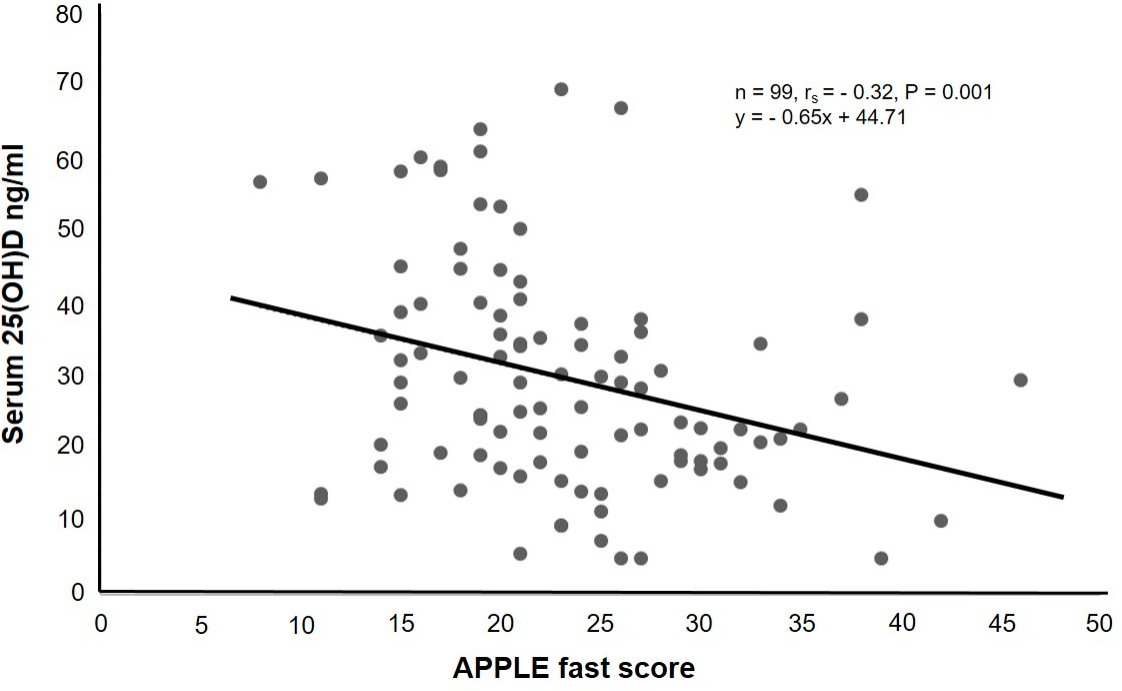
Correlation between serum 25(OH)D concentration and APPLE fast score. Serum 25(OH)D concentrations had a weak, inverse, yet significant correlation with APPLE fast scores.

The evaluation of the linear relationship between serum 25(OH)D concentrations and illness severity defined by APPLE fast scores resulted in an inverse, significant correlation (Fig 6).

### Serum 25(OH)D concentration and sepsis

**Fig 7:**
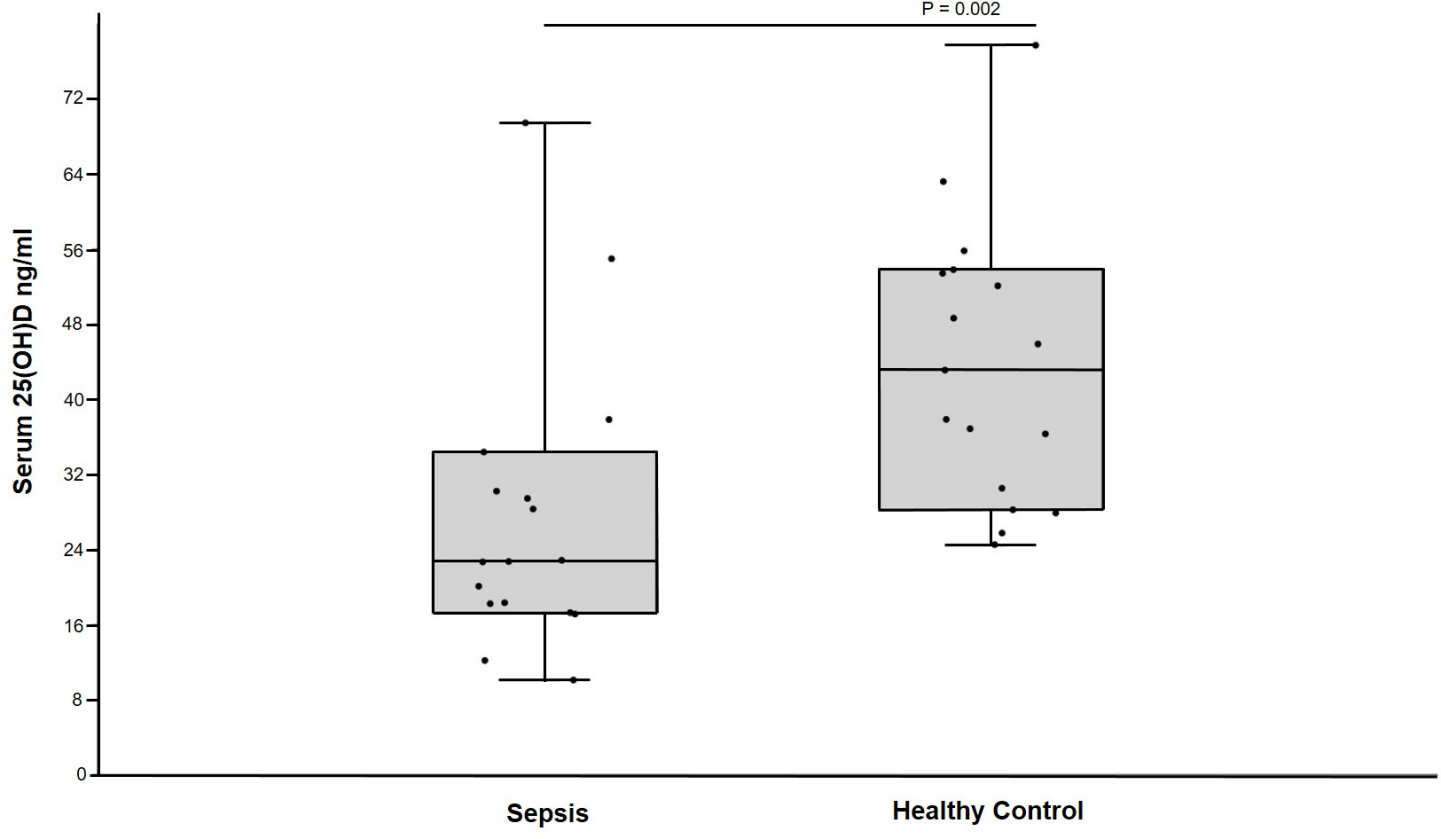
Box and whiskers plot comparing serum 25(OH)D concentrations in dogs with sepsis and healthy control dogs. The boxes represent the 25th and 75th quartiles with the horizontal line representing the median. The whiskers represent the range of the data. The black circles represent results for individual dogs. Healthy control dogs (n = 17) had significantly greater serum 25(OH)D concentration than dogs with sepsis (n = 17).

Dogs with sepsis had significantly lower serum 25(OH)D concentrations than healthy control dogs (Fig 7). There was not a significant difference (P = 0.67) in serum 25(OH)D concentration in dogs with sepsis that survived to discharge (n = 8 dogs, 25 ng/ml, 17, 37; 17–69) compared to those that died or were euthanized (n = 9 dogs, 22 ng/ml, 15, 29; 10–55).

## Discussion

In our investigation, we found that critically ill dogs had significantly lower serum 25(OH)D concentrations than healthy control dogs. Serum 25(OH)D concentration was an independent predictor of survival for dogs in the ICU as well as at 30 days after discharge. In addition, a serum 25(OH)D concentration cutoff of >33 ng/ml yielded a sensitivity of 94% and a specificity of 41% for predicting survival of critically ill dogs in our study. Serum 25(OH)D concentration had a significant correlation with illness severity but was not associated with length of stay in the ICU. Dogs with sepsis had significantly lower serum 25(OH) D concentrations than healthy control dogs but there was no difference in serum 25(OH)D concentration in dogs with sepsis that survived to discharge and those that died or were euthanized.

We found that serum 25(OH)D concentration was lower in critically ill dogs than in healthy control dogs. Furthermore, serum 25(OH)D was an independent predictor of survival in the ICU as well as at 30 days after discharge. This relationship is commonly found in critically ill people [1–9]. The pleiotropic effects of vitamin D in immunity, endothelial and mucosal functions as well as glucose and calcium homeostasis, may be fundamental to common illnesses encountered by critically ill dogs as they are in humans [42, 43]. More specifically, the role of vitamin D as a potent modulator of the immune system may best explain the increased risk of mortality in critically ill dogs and people. Vitamin D increases the innate immune response by enhancing phagocytosis [18–20] and induction of the antimicrobial peptides, cathelicidin and β-defensins [21–25]. Furthermore, immunologic production of calcitriol, the hormonally active form of vitamin D, down-regulated leukocyte production of pro-inflammatory cytokines such as IL-1, IL-2, TNF-α, and IL-6 while enhancing production of the anti-inflammatory cytokine IL-10 in humans [26–29]. Similarly, calcitriol blunts leukocyte production of TNF in dogs [30] and increases lipopolysaccharide stimulated leukocyte production of IL-10 in endotoxin primed whole blood in dogs, *in vitro* (Jaffey et al., unpublished work). Therefore, it is reasonable to postulate that during critical illness, the decreased circulating 25(OH)D reservoir needed for paracrine production of calcitriol is diminished resulting in compromised innate immune function and an exaggerated pro-inflammatory milieu.

The area under the ROC curve for serum 25(OH)D concentration in relation to ICU and 30 day survival was 0.70 and 0.72, respectively. The optimal cutoffs for ICU and 30 day survival were >33 ng/ml and >30 ng/ml yielding sensitivities and specificities for survival of 95%, 87% and 41%, 53%, respectively. These results indicate that serum 25(OH)D concentrations obtained within 24 hours of ICU admission has excellent sensitivity to detect survival in critically ill dogs. In contrast to its excellent sensitivity, serum 25(OH)D concentrations had a poor specificity for survival. In fact, only 1 of the 17 dogs that died or was euthanized in the ICU had a serum 25(OH)D concentration above 33 ng/ml. The only dog with a serum 25(OH)D concentration greater than the cutoff that died had acute fulminant liver failure. In people, liver dysfunction results in increased serum 25(OH)D concentrations [1, 44]. A conclusion of a recent meta-analysis was that vitamin D supplementation reduced mortality in critically ill people [45]. Additional studies are needed to assess if supplementation of vitamin D is associated with a survival benefit in critically ill dogs. Until those studies are available it is not recommended to apply our results in a clinical setting.

Length of stay in the ICU is associated with serum 25(OH)D concentrations in critically ill humans [1, 10]. This relationship makes sense because serum 25(OH)D concentration correlates well with illness severity in people. It stands to reason that more time and support is necessary to recover from a profound illness. Similarly, our study found that serum 25(OH)D concentration had a significant correlation with illness severity. However, there was not a significant correlation between serum 25(OH)D concentration and length of stay in our study. In addition, there was not a significant difference in serum 25(OH)D concentration when length of stay was dichotomized around the median (3 days) into 2 groups and compared. The exclusion of dogs that died or were euthanized from the length of stay analysis may have impacted our results.

Dogs with sepsis in our study had significantly lower serum 25(OH)D concentrations than did healthy control dogs. However, there was not a significant difference in serum 25(OH)D concentration in dogs with sepsis that survived to discharge and those that died or were euthanized. Our results are in contrast with numerous survival studies in humans with sepsis that indicate that hypovitaminosis D is predictive of ICU survival [8, 12, 29, 46, 47]. A limitation of this sub analysis is that it was performed with results from a small population of dogs with sepsis and may not have been powered to identify a difference if one had been present. Future, sufficiently powered studies are needed to investigate if serum 25(OH)D concentrations are independent predictors of survival in dogs with sepsis.

A number of limitations of our study should be considered when interpreting the results presented. Firstly, we cannot completely rule out the potential confounding influence of unassessed variables such as the volume infused in the initial hours after admission, vitamin D binding protein (VDBP) concentrations, and enteral nutrition. Intravenous fluid resuscitation in people lowers serum 25(OH)D concentration for up to 24 hours [48]. Additional studies are needed to know if fluid shifts within 24 hours of ICU admission influence serum 25(OH)D concentrations in critically ill dogs. We were unable to control for VDBP because a validated assay for use in dogs was not available during the time of our study. A decrease in this protein during critical illness could impact 25(OH)D concentrations. Vitamin D binding protein concentrations decrease in humans during times of diffuse inflammation, including in sepsis, severe trauma, and after surgery [49]. The association of vitamin D with its binding protein in humans is complex and non-linear [50]. Because of this complexity, future studies evaluating hypovitaminosis D and survival in dogs would benefit from also measuring VDBP concentrations. In addition, we did not investigate alternative vitamin D pathways or metabolites that may influence 25(OH)D concentrations or represent novel biomarkers for survival. An alternative vitamin D_3_ metabolism pathway utilizes steroidogenic enzyme cytochrome P450scc (CYP11A1) and 1α-hydroxylase (CYP27B1) to convert vitamin D_3_ to 20(OH)D_3_ and 1,20(OH)_2_D_3_, respectively, in people, pigs, and mice [51, 52]. Metabolites from this alternative pathway have comparable antiproliferative and anti-inflammatory properties as the classical 1,25(OH)_2_D_3_ system [52]. We did not record nutritional intake for the dogs in our study. Therefore, it is possible dogs provided assisted nutrition via tube feedings or voluntarily consumed food increased their serum 25(OH)D concentration. However, this is unlikely because the half time to equilibration of serum 25(OH)D for a given level of vitamin D intake is several weeks. Although serum 25(OH)D concentrations were obtained one time within 24 hours of admission, the duration of illness prior to sample collection varied. Vitamin D concentrations in people decrease in the acute phase of critical illness and increase during recovery [17]. It is possible that sampling at a different time point would have yielded different results. Lastly, we did not identify a causal organism in 4 dogs with sepsis. Two of these dogs had small intestinal foreign bodies with concomitant perforations identified at the time of surgery. The remaining 2 dogs were believed to have gut-derived sepsis [53].

## Conclusions

Our findings indicate that critically ill dogs and dogs with sepsis have significantly lower serum 25(OH)D concentrations than do healthy control dogs. In addition, serum 25(OH)D concentration obtained within 24 hours of hospital admission was an independent predictor of survival. Serum 25(OH)D concentration had excellent sensitivity, but poor specificity for detection of death. Serum 25(OH)D was inversely associated with APPLE fast score, but was not associated with ICU length of stay. Our results support the initiation of future clinical trials to investigate the effects of vitamin D (e.g., calcidiol or calcitriol) supplementation in critically ill dogs and indicates that dogs with severe illness may represent a valuable animal model to explore the effect vitamin D supplementation has on people in a critical care setting.
